# Supplementary material for: Zfp281 is essential for mouse epiblast maturation through transcriptional and epigenetic control of Nodal signaling
Source: eLife. 2017 Nov 23;6:e33333. doi: 10.7554/eLife.33333 (PMC5708896; doi:10.7554/eLife.33333)
Supplement: Transparent reporting form [file elife-33333-transrepform.pdf]

eLIFE
1st Floor
24 Hills Road
Cambridge CB2 1JP, UK
P 01223 855340
W elifesciences.org
T @elife

eLife's transparent reporting form

We encourage authors to provide detailed information *within their submission* to facilitate the interpretation and replication of experiments. If you have any questions, please contact us: editorial@elifesciences.org.

Sample-size estimation

- You should state whether an appropriate sample size was computed when the study was being designed
- You should state the statistical method of sample size computation and any required assumptions
- If no explicit power analysis was used, you should describe how you decided what sample (replicate) size (number) to use

Please outline where this information can be found within the submission (e.g., page numbers or figure legends), or explain why this information doesn't apply to your submission:

The number of embryos (or cells) used for immunostaining quantification and RNA-seq have been summarized in Legends of Figure 1 (and Figure 1-figure supplements), Figure 2 (and Figure 2-figure supplements), Figure 3 (and Figure 3-figure supplements), Figure 4 (and Figure 4-figure supplements).

Replicates

- You should report how often each experiment was performed
- You should include a definition of biological versus technical replication
- The data obtained should be provided and sufficient information should be provided to indicate the number of independent biological and/or technical replicates
- If you encountered any outliers, you should describe how these were handled
- Criteria for exclusion/inclusion of data should be clearly stated
- High-throughput sequence data should be uploaded before submission, with a private link for reviewers provided (these are available from both GEO and ArrayExpress)

Please outline where this information can be found within the submission (e.g., page numbers or figure legends), or explain why this information doesn't apply to your submission:

Embryo ChIP experiment was performed in two biological replicates (Legend of Figure 7). For all qPCR data, average expression was calculated with an error bar of standard error from two technique replicates. See Materials and Methods (line 560, 573).

Zfp281 ChIP-seq data and Zfp281KO embryo RNA-seq data are available in GEO under accession GSE93044.

eLife Sciences Publications, Ltd is a limited liability non-profit non-stock corporation incorporated in the State of Delaware, USA, with company number 5030732, and is registered in the UK with company number FC030576 and branch number BR015634 at the address 1st Floor, 24 Hills Road, Cambridge CB2 1JP | August 2014

Statistical reporting

- Statistical analysis methods should be described and justified
- Raw data should be presented in figures whenever informative to do so (typically when N per group is less than 10)
- For each experiment, you should identify the statistical tests used, exact values of N, definitions of center, methods of multiple test correction, and dispersion and precision measures (e.g., mean, median, SD, SEM, confidence intervals; and, for the major substantive results, a measure of effect size (e.g., Pearson's r, Cohen's d)
- Report exact p-values wherever possible alongside the summary statistics and 95% confidence intervals. These should be reported for all key questions and not only when the p-value is less than 0.05.

Please outline where this information can be found within the submission (e.g., page numbers or figure legends), or explain why this information doesn't apply to your submission:

For comparison of expression immunostaining levels, student T-test was used. And a p-value less than 0.05 was considered statistically significant. See Legends of Figure 1 (and Figure 1-figure supplements), Figure 2 (and Figure 2-figure supplements), Figure 3 (and Figure 3-figure supplements), Figure 4 (and Figure 4-figure supplements)

Phi-correlation was calculated for correlation of ChIP-seq data. See Materials and Methods (line 621-623) and Legend of Figure 6.

(For large datasets, or papers with a very large number of statistical tests, you may upload a single table file with tests, Ns, etc., with reference to page numbers in the manuscript.)

Additional data files ("source data")

- We encourage you to upload relevant additional data files, such as numerical data that are represented as a graph in a figure, or as a summary table
- Where provided, these should be in the most useful format, and they can be uploaded as "Source data" files linked to a main figure or table
- Include model definition files including the full list of parameters used
- Include code used for data analysis (e.g., R, MatLab)
- Avoid stating that data files are "available upon request"

Please indicate the figures or tables for which source data files have been provided:

Source data of Figure 2A has been uploaded as Supplement File 1.

eLife Sciences Publications, Ltd is a limited liability non-profit non-stock corporation incorporated in the State of Delaware, USA, with company number 5030732, and is registered in the UK with company number FC030576 and branch number BR015634 at the address 1st Floor, 24 Hills Road, Cambridge CB2 1JP | August 2014
